# Clinical and Laboratory Profile of Hospitalized Symptomatic COVID-19 Patients: Case Series Study From the First COVID-19 Center in the UAE

**DOI:** 10.3389/fcimb.2021.632965

**Published:** 2021-02-26

**Authors:** Suad Hannawi, Haifa Hannawi, Kashif Bin Naeem, Noha Mousaad Elemam, Mahmood Y. Hachim, Ibrahim. Y. Hachim, Abdulla Salah Darwish, Issa Al Salmi

**Affiliations:** ^1^ Department of Medicine, Ministry of Health and Prevention, Dubai, United Arab Emirates; ^2^ Department of Reserach, Ministry of Health and Prevention, Mohammed Bin Rashid University of Medicine and Health Sciences, Dubai, United Arab Emirates; ^3^ Sharjah Institute for Medical Research, College of Medicine, University of Sharjah, Sharjah, United Arab Emirates; ^4^ Clinical Sciences Department, College of Medicine, University of Sharjah, Sharjah, United Arab Emirates; ^5^ College of Medicine, Mohammed Bin Rashid University of Medicine and Health Sciences, Dubai, United Arab Emirates; ^6^ Gullf Medical School, Ajman, Gulf Medical University, Ajman, United Arab Emirates; ^7^ Department of Medicine, Oman Medical Specialty Board, The Royal Hospital, Muscat, Oman; ^8^ The Research Section, Oman Medical Speciality Board, The Royal Hospital, Muscat, Oman

**Keywords:** COVID-19, acute respiratory distress syndrome, clinical characteristics, laboratory features, organ failure, mechanical ventilation

## Abstract

**Introduction:**

COVID-19 is raising with a second wave threatening many countries. Therefore, it is important to understand COVID-19 characteristics across different countries.

**Methods:**

This is a cross-sectional descriptive study of 525 hospitalized symptomatic COVID-19 patients, from the central federal hospital in Dubai-UAE during period of March to August 2020.

**Results:**

UAE’s COVID-19 patients were relatively young; mean (SD) of the age 49(15) years, 130 (25%) were older than 60 and 4 (<1%) were younger than 18 years old. Majority were male(47; 78%). The mean (SD) BMI was 29 (6) kg/m^2^. While the source of contracting COVID-19 was not known in 369 (70%) of patients, 29 (6%) reported travel to overseas-country and 127 (24%) reported contact with another COVID-19 case/s. At least one comorbidity was present in 284 (54%) of patients and 241 (46%) had none. The most common comorbidities were diabetes (177; 34%) and hypertension (166; 32%). The mean (SD) of symptoms duration was 6 (3) days. The most common symptoms at hospitalization were fever (340; 65%), cough (296; 56%), and shortness of breath (SOB) (243; 46%). Most of the laboratory values were within normal range, but (184; 35%) of patients had lymphopenia, 43 (8%) had neutrophilia, and 116 (22%) had prolong international normalized ratio (INR), and 317 (60%) had high D-dimer. Chest x ray findings of consolidation was present in 334 (64%) of patients and CT scan ground glass appearance was present in 354 (68%). Acute cardiac injury occurred in 124 (24%), acute kidney injury in 111 (21%), liver injury in 101 (19%), ARDS in 155 (30%), acidosis in 118 (22%), and septic shock in 93 (18%). Consequently, 150 (29%) required ICU admission with 103 (20%) needed mechanical ventilation.

**Conclusions:**

The study demonstrated the special profile of COVID-19 in UAE. Patients were young with diabetes and/or hypertension and associated with severe infection as shown by various clinical and laboratory data necessitating ICU admission.

## Introduction

Corona virus disease -2019 (COVID-19) caused by severe acute respiratory syndrome coronavirus 2 (SARS-CoV-2), was first detected in Wuhan-China on December 31st, 2019 (Wang C et al., 2020). Shortly after, the World Health Organization (WHO). declared COVID-19 as a global pandemic on March 2020 (Mahase, 2020). As on January 19^th^, 2021 the total global number of affected cases was 96,073,719 million and 2,051,377 deaths Available at: https://www.worldometers.info/coronavirus/. In UAE A Chinese family on holiday in the UAE were the first people in the country to be given positive coronavirus diagnoses on January 23^rd^, 2020 (Zaatar, ), Thereafter, the cases increased to reach 63,819 as on August 14^th^ 2020 (The Supreme Council for National Security, ).

SARS-CoV-2 is highly contagious (Mohapatra et al., 2020) and one of way to control the pandemic is through preventive methods, sensitive diagnostic approaches, and using accessible drugs, in a view of understanding the characteristics of COVID-19 among different population (Lotfi et al., 2020).

Although the majority of the patients have mild symptoms, COVID-19 can be fatal and require intensive medical care (Sheleme et al., 2020). COVID-19 severity and fatality have been found to be associated with many host factors, including age, gender, race, ethnicity and presence of other comorbidities (Biswas and Mudi, 2020). Moreover, it had been reported that COVID-19 mortality rates differ between countries and between geographical areas within the same country with genetic factors and climates differences as an etiology for this variation (Biswas and Mudi, 2020; Al-Tawfiq et al., 2020). Report showed that Asian individuals had an increased risk of infection and worse clinical outcomes and higher mortality (Pan et al., 2020). Therefore, this study aimed at studying the clinical and the laboratory characteristics of symptomatic hospitalized COVID-19 patients that had been admitted to the main federal hospital in Dubai, United Arab Emirates (UAE). Understanding COVID-19 profile in each country will help in containing the disease and to set proper guidelines and strategies to deal with any subsequent waves.

## Methods

### Study Design

This clinical observational study included 525 hospitalized symptomatic confirmed COVID-19 patients admitted to Al Kuwait-Dubai hospital (AKH), Dubai, United Arab Emirates (UAE) between February until August, 2020. The AKH is the only federal hospital operated by the Ministry of health and Prevention (MOHAP) in Dubai, and it is the first hospital in the UAE that had been evacuated from all other cases and had been declared as a COVID-19 center. Up to the date of writing this manuscript, AKH is still function as a COVID-19 center.

### Data Collection

COVID-19 had been confirmed using polymerase chain reaction (PCR) from nasal swab sample. The laboratory used Sacace Real Time Reverse Transcription Polymerase Chain Reaction (rRT-PCR) test to diagnose COVID-19 disease. The test was performed on patients’ nasopharyngeal swabs. RNA was extracted using SaMag Viral Nucleic Acid Extraction system. Extracted RNA was amplified using BGI-Real Time Fluorescent RT-PCR kit for the detection of COVID-19. Suspected cases were admitted to a different specialized hospital devoted for unconfirmed cases.

MOHAP hospitals use electronic medical file system where all clinical, laboratory, radiological and management data are collected prospectively.

Collected data included (1) demography; age, gender, body mass index (BMI); calculated as weight(KG)/height(m^2^), (2) source of infection; travel to overseas, contact with another COVID-19 patient, or unknown source (identified as either no travel history and no contact with another known positive case of COVID-19), (3) smoking status (current or ex-smoker), (4) comorbidities; diabetes mellitus (DM), hypertension, cardiovascular disease (CVD), chronic lung disease, chronic kidney disease (CKD), stroke/transient ischemic attack (TIA), cancer, and any other documented comorbidities (5) COVID-19 symptoms; (A) duration of symptoms (days), (B) upper respiratory tract infection symptoms; headache, fever, fatigue, myalgia, rhinorrhea, and sore throat (C) lower respiratory tract infection symptoms; cough, shortness of breath (SOB), sputum production, and hemoptysis, (D) other symptoms; ageusia, anosmia, anorexia, nausea, vomiting, diarrhea, and confusion, (6) radiological features; chest X-ray consolidation and computed tomography (CT) ground glass appearance, (7) clinical complications such as (A) presence of organ failure; (i) acute cardiac injury (defined as at least one documented elevated high-sensitivity troponin-I) (ii) acute kidney injury; defined as a raise in the serum creatinine of ≥26.5 µmol/liter within 48 h.; or increase in serum creatinine ≥1.5 times than the baseline that occurred within the preceding 7 days; or if there were reduction in the urine volume <0.5 ml/kg/hr. for 6 consecutive h, and (iii) acute liver injury; defined as presence of high alanine transaminase (ALT) and/or high aspartate aminotransferase (AST) by more than 5 times the upper limit of normal range, (iv) acute respiratory distress syndrome (ARDS); using Berlin definition (Force et al., 2012), acidosis, (5) septic shock; defined as per the Third International Consensus Definitions for Sepsis and Septic Shock (Singer et al., 2016), (6) need for intensive care unit (ICU) medical care, (7) need for mechanical ventilation, and (8) death rate.

We followed the Chinese CDC report in categorizing the COVID-19 clinical manifestations to 3 levels of severity; (1) Mild disease; if there were non-pneumonia or if there were mild pneumonia, (2) Severe if there were dyspnea, respiratory rate ≥ 30/min, blood oxygen saturation (SpO2) ≤ 93%, PaO2/FiO2 ratio [the ratio between the partial pressure of oxygen (PaO2) and the fraction of inspired oxygen (FiO2)] < 300, and/or lung infiltrates > 50% within 24 to 48 h, and (3) Critical, if there were respiratory failure, septic shock, and/or multiple organ dysfunction/failure (Wu and McGoogan, 2020).

The medications used in treating COVID-19 during their admission are as follow: Chloroquine/Hydroxychloroquine, Lopinavir/Ritonavir, antibiotics, Steroid, Interferon, Tocilizumab, and anti-fungal.

The laboratory tests were collected and include: (1) blood rheology; hemoglobin (Hb) (normal reference range; NR: male: 12.0-15.5 g/dl, and female: 13.5 -17.5 g/dl), total white cell count (WCC) (NR: 4,000 -11,000 x10(3)/mcl), lymphocyte count (NR: 1,000 and 4,800 x10(3)/mcl), neutrophil count (NR: 1.5-4 x10(3)/mcl), and platelet count (NR: 150,000 - 400,000 x10(3)/mcl), (2) inflammatory markers; ferritin (NR: males: 26-388 ng/mL, and female: 8-252 ng/mL), C-reactive protein (CRP) (NR: 0.0-5.0 mg/liter), procalcitonin (NR < 0.1 ng/mL), lactate dehydrogenase (LDH) (NR: Male: 85 – 227 U/liter, and Female: 81 – 234 U/liter) and lactic acid (NR: 0.4 – 2.0 mmol/liter), (3)glucose status; glycosylated hemoglobin; HbA1C (NR: 4.8 - 6.0%), (3) liver function test; ALT (NR: males: 16 – 63 IU/liter, and females:14 – 59 IU/liter), AST (NR: 15 – 37 U/liter), albumin (34 – 50 g/liter), bilirubin (NR: 0-3 µmol/liter), and alkaline phosphatase level (NR: 46-116 U/liter) (4) renal function test, included, blood urea (NR: 0.0-8.3 mmole/liter), and creatinine (NR: 44-133 µmole/liter). Estimated glomerular filtration rate (eGFR) (NR: 90 to 120 mL/min/1.73 m^2^) had been calculated using Modification of Diet in Renal Disease (MDRD) equation, 186 × (SCr mg/dl)-1.154 × (age)−0203 × 0.702 [if Female] x 1.212[if Black], (5) Coagulation profile included International normalized ratio (INR) (NR <1.1), and D-dimer (>55 mg/liter considered positive) (6) electrolytes included sodium (Na) level (NR: 135-145 mEq/liter) and potassium (K) level (NR: 3.6 to 5.2 mmol/liter), and (7) indicator of cardiac injury; troponin level (NR: 0 to 60.4 ng/liter).

Further, laboratory parameters had been categorized based on the upper and lower limits of the normal reference ranges to lymphopenia if lymphocyte count was < 1 x10^3^/mcL, neutrophilia if neutrophil counts were >14 x10(3)/mcL, high ALT if were > 63 IU/liter for male and >59 IU/liter for female, high AST if were > 37 U/liter, high LDH if were > 227 IU/liter for males and > 234 IU/liter for females, high ferritin if were > 388 ng/ml for males and were > 252 ng/ml for female, high D-dimer if were > 0.5 mg/liter, high troponin if were > 60 ng/liter, high lactic acid if were >2.0 mmol/liter, and high procalcitonin if were > 0.1 µg/liter.

### Statistical Analysis

Data had been presented as mean and standard deviation for continuous variables and frequency (number and percentage; %) for categorical variables. To assess the differences between COVID-19 patients needed ICU admission vs. no ICU admission Student’s t-test was used for the continuous variables and Chi-square test was used for the categorical variables. P value <0.05 had been considered significant. All the analysis had been carried out using STATA 9/SE statistical software (Stata Corp, College Station, Texas, USA).

### Ethical Approval

The study was approved by the Scientific Research Committee. Approval Number. MOHAP/DXB-REC/MMM/NO.44/2020 and certify that the study was performed in accordance with the ethical standards as laid down in the 1964 Declaration of Helsinki and its later amendments ethical standards.

## Results

### Demography

The basic clinical and epidemiological features of this cohort is shown in **Table 1**. While the majority of the patients were from the indicant subcontinent; 332 (63%), the UAE citizens made 53 (10%) and the other Arabs were 75 (14%). The mean (SD) of the age was 49 ± 15 years, with only 130 (25%) of the patient were older than 60 years old, and 4 (0.76%) were younger than 18 years old. Male composed the majority of the patients with ratio of female to male was 118: (22%) to 407 (78%). The mean (SD) of the BMI was 29 ± 6 kg/m^2^ for all COVID 19 patients.

**Table 1:** Demography of 525 symptomatic COVID-19 patients.

Variables	
**Nationalities**	
United Arab Emirates	53 (10%)
Other Arabs	75 (14%)
Iranian	10 (2%)
Indian Subcontinent	332 (63%)
South East Asia	35 (7%), out of which 2 (0.4%) were Chinese
African	5 (1%)
European	13 (2.5%)
South American	2 (0.4%)
Age*	49 (15)
Age > 60 yrs.	130 (25%)
Age <18 yrs.	4 (0.76%)
Gender (Females: Males)	118: (22%): 407 (78%)
BMI (kg/m^2^)*	29 (6)
Smokers (current or ex-smoker)	25 (5%)
Symptoms duration (days)	6 (3)*
**Source of COVID-19 infection**	
Travel history	29 (6%)
Contact history	127 (24%)
No known source for COVID-19	369 (70%)

Data presented as number (%) or as mean (SD; standard deviation)*, >; more than, <; less than.

The source of infection transmission was unknown for the majority of the patients 369 (70%) with only 29 (6%) and 127 (24%) had history of travel to foreign overseas country and history of contact with another COVID-19 patient, respectively. Smoking history; either current smoking or history of smoking presented in 25 (5%) of patients. At presentation to the hospital the mean (SD) of COVID-19 symptoms duration was 6 ± 3 days.

### Comorbidities

While 284 (54%) of the patients had at least one comorbidity, 241 (45%) had none. The most common comorbidities were DM (177; 34%) and hypertension (166; 32%). CVD was present in 27 (5%) of patients. Small percentage of patients had thyroid dysfunction (16; 3%), chronic lung disease (14; 3%), and chronic kidney disease (13; 2%), as shown in **Table 2**.

**Table 2:** Comorbidities of 525 symptomatic COVID-19 patients.

Comorbidities	No		Yes	
	n	%	n	%
Presence of any comorbidities	241	45.90	284	54
Diabetes mellitus	348	66	177	34
Hypertension	359	68	166	32
Cardiovascular disease	498	95	27	5
Thyroid dysfunction	509	97	16	3
Chronic lung disease	511	97	14	3
Chronic kidney disease	512	98	13	2
Stroke	521	99.24	4	0.76
Cancer	522	99.43	3	0.57
Epilepsy	524	99.8	1	0.2
Multiple sclerosis	524	99.8	1	0.2
Autoimmune rheumatic diseases	523	99.6	2	0.4
Thalassemia	524	99.8	1	0.2

Data presented as n; number and %; percentage.

### Clinical Symptoms

**Figure 1:**
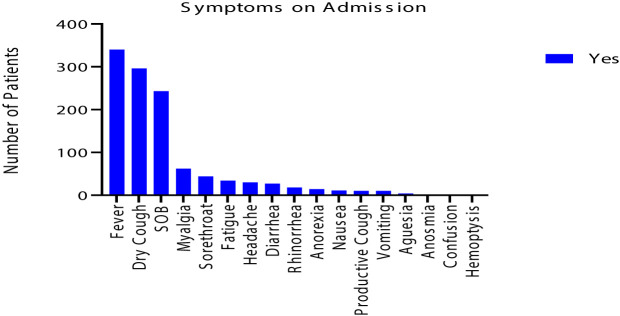
The distribution of the 525 symptomatic COVID-19 patients on admission.

The most common symptoms were fever (340; 65%), cough (296; 56%), and SOB (243; 46%). The cough was mainly dry with only 10 (2%) had sputum production with no hemoptysis. Small percentage of patients had myalgia (62; 12%), sore throat (44; 8%), headache (30; 6%) and fatigue (34; 6%). Less than and/or equal to 5% of patients had anorexia (14; 3%), rhinorrhea (18; 3%), nausea (11; 2%), vomiting (10; 2%), and diarrhea (27; 5%). 2 (0.4%) of patients had anosmia, and 4(1%) had ageusia. None of the patients had confusion, as shown in **Table 3** and in **Figure 1**.

**Table 3:** Symptoms of 525 symptomatic COVID-19 patients.

Symptoms	NO		YES	
	n	%	n	%
Fever	185	35	340	65
Cough	229	44	296	56
Fatigue	491	94	34	6
Anorexia	511	97	14	3
Shortness of breath	282	54	243	46
Sputum production	515	98	10	2
Myalgia	463	88	62	12
Headache	495	94	30	6
Confusion	0	0	0	0
Rhinorrhea	507	97	18	3
Sore throat	481	92	44	8
Hemoptysis	0	0	0	0
Vomiting	515	98	10	2
Diarrhea	497	95	27	5
Nausea	514	98	11	2
Anosmia	523	99.6	2	0.4
Ageusia	521	99	4	1

Data presented as n; number and %; percentage.

### Laboratory Parameters and Radiological Features

While most of the blood rheology parameters values (Hb, WCC, and platelet count) were within normal range at time of first encounter to the hospital,184 (35%) of the patients had lymphopenia and 43 (8%) had neutrophilia. The coagulation profile showed 116 (22%) of the patients had prolonged INR, and 317 (60%) had high D-dimer.

**Figure 2:**
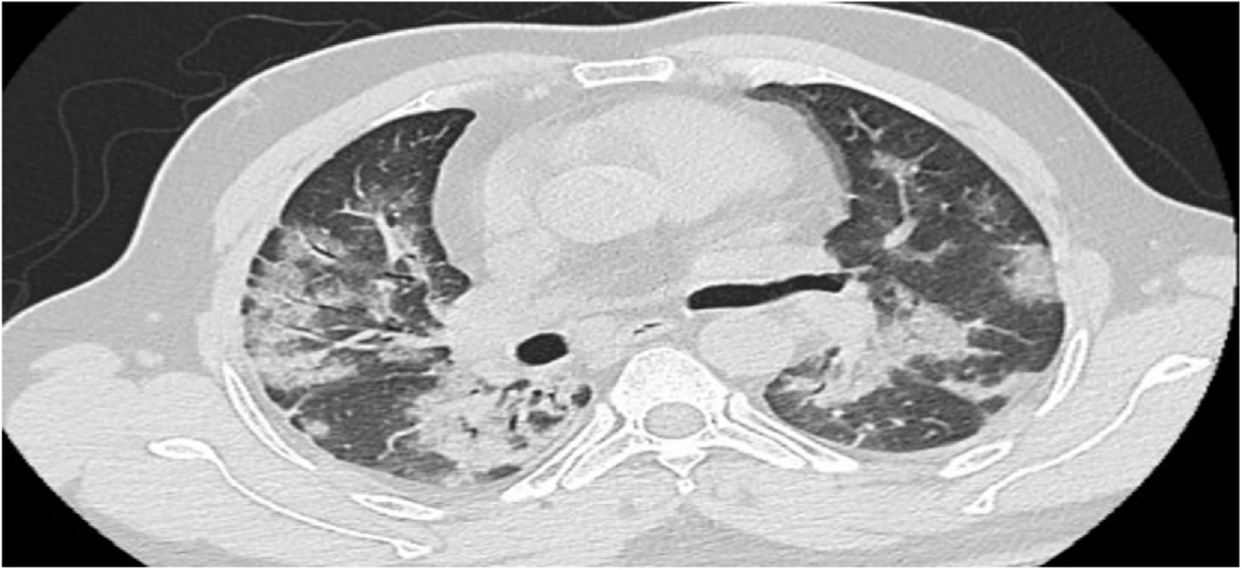
Bilateral multilobar ground-glass opacification and consolidations, preferentially peripheral and subpleural.

Radiological investigations at admission showed bilateral chest x ray consolidation was present in 334 (64%) of patients and CT scan ground glass appearance was present in 354 (68%), as shown in **Table 4** and **Figure 2**.

**Table 4:** Laboratory features of 525 symptomatic COVID-19 patients.

	mean	SD
**Complete blood count**		
WCC [×10(3)/mcl]	8.44	4.05
Neutrophils count, [×10(3)/mcl]	6.39	3.88
Neutrophilia >14 [×10(3)/mcl], n(%)*	43 (8%)	
Lymphocytes count, [×10(3)/mcl]	1.36	0.74
Lymphocytes <1 [×10(3)/mcl] n (%)*	184 (35%)	
Hb (g/dl)	13	2
Platelet,[×10(3)/mcl]	250	102
**Coagulation profile**		
INR	1.07	0.14
Prolong INR*	116 (22%)	
D-dimer (mg/liter)	2.67	5.97
High D-dimer, n(%),(mg/liter)*	317 (60%)	
**Electrolytes and renal profile**		
Sodium (Na) (mmole/liter)	136.29	4.72
Potassium (K) (mmole/liter)	4.06	0.64
Urea (mmole/liter)	6.27	5.67
Creatinine (µmole/liter)	117.63	317.32
eGFR (ml/min)	86.00	32.16
**Liver function test**		
Serum bilirubin (µmol/liter)	16.03	39.21
ALT (IU/liter)	67.76	120.38
AST (U/liter)	58.85	116.11
High ALT/AST (IU/liter/U/liter), n(%)*	281 (54%)	
Alkaline phosphatase (IU/liter)	88.34	54.14
Albumin (g/liter)	30.77	7.21
**Inflammatory markers**		
CRP mg/liter	82.73	97.53
High CRP>3mg/liter, n(%)*	447 (85%)	
Procalcitonin (ug/liter)	0.82	4.03
High procalcitonin, n(%),(ug/liter)*	206 (39%)	
LDH (IU/liter)	417.834	323.13
High LDH, n(%),(IU/L)*	393 (75%)	
Lactic acid (mmole/liter)	1.62	0.89
High lactic acid, mmole/liter, n(%)*	49 (9%)	
High ferritin, n(%),(mcg/liter)*	291 (55%)	
Ferritin (mcg/liter)	1049.50	1429.78
**Other parameters**		
Troponin (ng/liter)	278.62	2744.61
High troponin (ng/liter)*	69 (13%)	
HbA1C (%)	7.08	2.41

Data presented as mean and SD; or as n; number (%; percentage)*, standard deviation, WCC; white cell count, Hb; hemoglobin, INR; International normalized ratio, eGFR; estimated glomerular filtration rate, ALT; alanine transaminase, AST; aspartate aminotransferase, CRP; C-reactive protein, LDH; lactate dehydrogenase, HbA1C; glycosylated hemoglobin.

### Clinical Complications and Outcome

**Figure 3:**
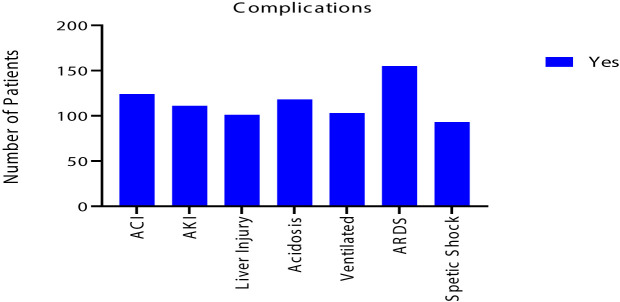
Clinical complications of the 525 symptomatic COVID-19 patients.

**Figure 4:**
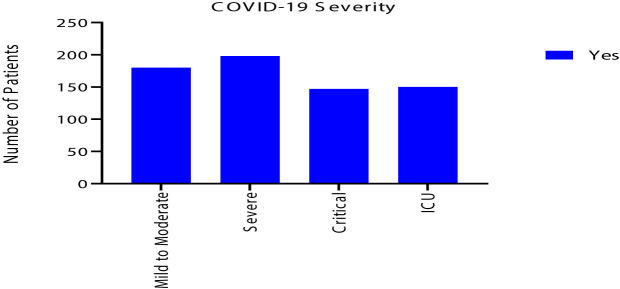
Clinical severity of the 525 symptomatic COVID-19 patients.

Despite that most of renal and liver function tests were within normal range, 111 (21%) had acute kidney injury and 101 (19%) had liver injury. Also, 124 (24%) of COVID-19 patients had acute cardiac injury. In addition, ARDS developed in 155 (30%), acidosis in 118 (22%) and septic shock in 93 (18%) of patients. As a result, 150 (29%) required ICU admission with 103 (20%) needed mechanical ventilation. Eventually, 93 (18%) of the patients deceased, as shown in **Table 5** and **Figures 3** and **4**.

**Table 5:** Clinical complications and clinical outcomes of 525 symptomatic COVID-19 patients.

Clinical complications and outcomes	No		YES	
	n	%	n	%
**Severity**				
Mild to moderate			181	34
Sever			197	38
Critical			147	28
**Radiological features**				
Bilateral chest X-ray consolidation	191	36	334	64
CT ground glass appearance	171	33	354	68
**Organ failures and complications**				
Acute cardiac injury	401	76	124	24
Acute kidney injury	414	79	111	21
Liver injury	424	81	101	19
Acidosis	407	78	118	22
Septic shock	432	82	93	18
ARDS	370	70	155	30
Mechanical ventilation	422	80	103	20
ICU admission	375	71	150	29
Death	432	82	93	18

Data presented as n; number and %; percentage, CT; computed tomography, ARDS; acute respiratory distress syndrome, ICU; intensive care unit.

### Medications Used in the Treatment of COVID-19 Patients

Drugs used to treat patients as per National (UAE) Guidelines for Clinical Management and Treatment of COVID-19. 20 April 2020, version 3.1. The guidelines recommend different classes of drugs according to the clinical status. For laboratory-confirmed COVID-19 patients without pneumonia, hydroxychloroquine/chloroquine with lopinavir-ritonavir is recommended. In the presence of radiological evidence of pneumonia, favipiravir and remdesivir is considered additionally. In case of critical illness, tocilizumab and interferon-alfa 2 is considered additionally. Steroids are used in the presence of significant hypoxia with high inflammatory markers. Antibiotics are added when a super-added bacterial infection is suspected either clinically or positive cultures with high lab markers like CRP and procalcitonin. Antifungals are used if clinical suspicion of fungal infection or positive cultures.

This study showed that Chloroquine or hydroxychloroquine were the most commonly used medications in 493 (94%) of patients, followed by anti-viral; lopinavir/ritonavir in 380 (72%). Another anti-viral treatment used was Favipiravir which was used in 181 (34%) of patients. Anti-biotics had been prescribed in 372 (71%) of patients. Steroid used in over half of the patient; 298 (57%). Interferon, Tocilizumab (IL-6 inhibitor) and anti-fungal medications had been used in a smaller number of cases; 137 (26%), 105 (20%), and 59 (11%), respectively, as shown in **Table 6**.

**Table 6:** Treatment of 525 symptomatic COVID-19 patients.

Medications	No		YES	
	n	%	n	%
Chloroquine/hydroxychloroquine	32	6	493	94
Lopinavir/ritonavir	145	28	380	72
Favipiravir	344	66	181	34
Anti-biotics	153	29	372	71
steroid	227	43	298	57
Interferon	388	74	137	26
Tocilizumab (Interlukin-6; IL-6 inhibitor)	420	80	105	20
Anti-fungal	466	89	59	11

Data presented as n; number and %; percentage.

### COVID-19 Patients Needed ICU Care vs. COVID-19 Patients Did Not Need ICU Care

Among all the COVID-19 patients 150 (29%) required ICU admission without any prediction for any nationality to be admitted to the ICU. COVID-19 patients who needed ICU admission were older in age 55 ± 13 vs. 46 ± 15 years (p < 0.001), with 53 (35%) were older than 60 years compared to 77 (21%) of patients who did not required ICU care. Although male gender was the majority of COVID-19 patient but the percentage was higher among the group that required ICU care; 139 (93%) vs. 268 (21%), p < 0.001. More, they were more diabetic; 79 (53%) vs. 98 (26%) and hypertensive; 74 (49%) vs. 92 (25%) (p value < 0.001 for both) (**Tables 7** and **8**).

**Table 7:** Nationalities of the 525 symptomatic COVID-19 patients and admission to the intensive care unit (ICU).

	Total number of cases	ICU care (no)	ICU care (yes)
UAE nationals	53	42 (79%)	11 (21%)
Other Arabs	75	55 (73%)	20 (27%)
Iranian	10	8 (80%)	2 (20%)
Indian subcontinents	332	226 (68%)	106 (32%)
Chinese	2	2 (100%)	0 (0%)
African	5	5 (100%)	0 (0%)
European	13	12 (92%)	1 (8%)
South East Asian	33	23 (70%)	10 (30%)
South America	2	2 (100%)	0 (0%)

Data presented as number (%). P value 0.24.

**Table 8:** Age, gender, body mass index, symptoms duration and comorbidities of 150 symptomatic COVID-19 patients needed Intensive care unit (ICU) admission vs. 375 did not need ICU admission.

	ICU care (no) (n = 375)	ICU care (yes) (n = 150)	P value
Age (years)*	46 (15)	55 (13)	<0.001
Age > 60 years	77 (21%)	53 (35%)	<0.001
Gender (male: female)	268 (71%): 107 (29%)	139 (93%):11 (7%)	<0.001
BMI (kg/m2)*	29 (6)	29 (5)	0.878
Symptoms duration*	5.6 (3)	5.7 (3)	0.735
**Comorbidities**			
Cardiovascular disease	15 (4%)	12 (8%)	0.061
Diabetes	98 (26%)	79 (53%)	<0.001
Hypertension	92 (25%)	74 (49%)	<0.001
Stroke	3 (0.8%)	1 (0.7%)	0.874
Cancer	2 (0.5%)	1 (0.7%)	0.855
Chronic lung disease	9 (2%)	5 (3%)	0.549
Chronic kidney disease	8 (2%)	5 (3%)	0.424
Smoking history	17 (5%)	8 (5%)	0.697
Thyroid disease	12 (3%)	4 (3%)	0.748

Data presented as number (%) or as mean (SD; standard deviation)*.

## Discussion

To the best of our knowledge, this is the largest study from our region of the Gulf Cooperation Council (GCC) to examine both the clinical and laboratory features of admitted COVID 19 patients. It showed that patients were young, mostly male and overweight or obese. Majority were teetotal and their infection source was unknow in majority of patients with average symptoms duration of 6 days. More than 50% had either diabetes or hypertension or both as a medical comorbidity. Majority had typical LRTI symptoms and signs at presentation. Also, radiological findings were very common including ground glass appearance. Multi organ failure was noted and mostly include AKI, ALI, and ACI with almost a third needed ICU management. Management strategies were instituted as experience progressed with majority received including Chloroquine or hydroxychloroquine, anti-viral, antibiotics, steroids, and various biological medications.

The excess number of males in our sample is ongoing with what had been reported of increase vulnerability of men to COVID-19. Bwire et al. attributed this to the different ability of each gender to fight SARS-2-CoV-2 as a result of biological differences in the immune systems between men and women (Bwire, 2020). As well, it had been reported that females are more resistant to infections than men, and this is mediated by several factors including sex hormones and high expression of coronavirus receptors (ACE 2) in men (Bwire, 2020). More, life style differences between men and women such as smoking and drinking that are more common among men. As well, women have more responsible attitude toward the Covid-19 pandemic than men. Others reported that as men show higher mortality from diseases including heart disease and diabetes, then these diseases which are known to show sex-specific occurrence could be contributing factors for the sex-biased mortality from COVID-19 (Pradhan and Olsson, 2020).

Despite older age had been related to worse clinical outcomes in COVID-19 (Liu Y et al., 2020), the average age of our COVID-19 showed that they are relatively young (37.0 ± 13.0 years) compared to what had been reported in Oman (Khamis et al., 2020) and 41 years in Kuwait (Singh, 2020). As well, there were only 4 (0.76%) of our study patients below the age of 18 years which is close to what had been observed from the first cases reported in Wuhan where they reported no clinical cases in children below 15 years of age (Li et al., 2020). Further, other reports showed that COVID-19 appears to be less severe in children (Rezaei, 2020). UAE is a young nation with majority of its population between age 25 to 54 years (Statistic center D, ).

A systemic review demonstrated that proportion of male patients ranged from 29.0% to 77.0 (Fu et al., 2020). Other studies showed that there were no significant gender differences in prevalence of COVID-19 (Li et al., 2020), but men are more at risk for worse COVID-19 outcomes and death, independent of their age (Jin et al., 2020). In our study the majority of cases were male; 407 (78%). This could be explained by having in our study symptomatic COVID-19 patients who had been admitted to the hospital, therefore, our sample sized represent the worst sector of patients who required medical care for their disease.

Among our patients 5% had a history of smoking; either current or past. This is going with what had been reported in systemic review that the proportion of COVID-19 patients who were current smokers ranged from 0.0% to 18.0% (median 7.2%; nine studies) (Fu et al., 2020). Further, smoking is culturally not accepted in our region and is faced by a stigma in a conservative culture (Saravanan et al., 2019; Ayalon, 2019). Or, because of the stigma patients were not truthful revealing their smoking status.

The mean BMI of COVID-19 patients were 29 ± 6 kg/m^2^, which lies within the overweight class as per the WHO classification (Pi-Sunyer, 2000). Population and patients with high BMI have moderate to high risk of medical complications with COVID-19 (Malik et al., 2020), with increased adiposity destabilizes the pulmonary function and contribute to viral pathogenesis (Dorner et al., 2010).

The source of infection was known in about quarter of our COVID-19 patients while the majority of our symptomatic patients had no known source of infection. The unknown source of infection in the majority of our patient is in line with our previous finding (under review) that highlighted the adopted policy of paying attention to patients with travel or contact history resulted in unintentional negligence of other COVID-19 patients who had no history of travel or history of contact with another COVID-19 case. This strategy contributed to a worse clinical outcome among the COVID-19 patients with no obvious source of contracting the disease.

Additionally, the unknown source of infection can be explained by the transmission efficiency of SARS-CoV-2 that had proved to be high (Fauci et al., 2020). An infected individual can release aerosols and droplets containing SARS-CoV-2 by coughing, sneezing, speech and breathing (Chao et al., 2009; Tang et al., 2011). More, Aerosols (< 10-μm diameter) and droplets (> 10-μm diameter) can promote infection through deposition on surfaces and subsequent hand-to-mouth/nose/eye transfer, and through inhalation (van Doremalen et al., 2020).

Previous publications from other parts of the world revealed that pre-existing conditions (e.g., cardiovascular, pulmonary, and renal diseases) render a person more vulnerable to more severe COVID-19 infection (Zhou et al., 2020). Among our patient the most common comorbidities were DM and hypertension. Indeed, DM, hypertension and obesity are the most prevalent comorbidities in the UAE and its neighboring countries (Alharbi et al., 2014; Tailakh et al., 2014).

The most common symptoms among our patients were fever (65%), cough (56%), and SOB (46%). This is contradicting the results of a systemic review which showed fever, sore throat, and muscle soreness or fatigue as the most common symptoms (Sun et al., 2020). This might raise the alert that the manifestations of COVID-19 vary across different countries and different nations. Therefore, in our region more attention might be needed to be paid to LRTI symptoms than to the URTI symptoms.

In the early stage of COVID-19, decreased lymphocyte count had been reported and had been demonstrated as a negative prognostic factor (Cascella et al., 2020). The presence of lymphopenia had been seen in more than third of our patients; precisely in 35%. The relatively high procalcitonin level in 39% of our patients might indicate the presence of secondary bacterial infection. Presence of high CRP in 85%, high LDH in 75%, and high ferritin in 55% going with the inflammatory status of the disease. As well, presence of the high ALT, AST, and troponin level is aligned with the presence of the underlying organ dysfunction.

Although at early stage of COVID-19, the chest x ray might be normal, the presence of bilateral changes in 334 (64%) of our COVID-19 patients could be because of presence of patients to the hospital after 6 days of disease onset. And hence the CT scan ground glass appearance was seen in 354 (68%) of the patients. Yet, the CT changes are less than what had been reported in other studies where it was found to be 96.6% (Sun et al., 2020).

Up to the time of writing this manuscript, there is no specific antiviral treatment recommended for COVID-19. Yet, several medications have been proposed such as Lopinavir/Ritonavir (Bimonte et al., 2020) that we used in 94% of our patients. Despite that Lopinavir/Ritonavir showed some benefit is some studies, other demonstrated no benefit with Lopinavir/Ritonavir treatment compared to standard care (Cao et al., 2020). Favipiravir is another anti-viral agent that demonstrated efficacy, improve the discharging rate and decrease the mortality rate of COVID-19 patients (Wang Z et al., 2020), and had been used in 34% of our patients. Chloroquine and hydroxychloroquine were proposed as immunomodulatory therapy in COVID-19 and it was the most commonly used treatment in our patients; 94%. Chloroquine/hydroxychloroquine had been used massively in our patients despite the conflict results about its efficacy, with some studies support its use (Liu J et al., 2020) and other studies antagonize it use in COVID-19 (Kim et al., 2020). Corticosteroids is another drug that introduced as COVID-19 therapy; corticosteroid dexamethasone was suggested to have anti-inflammatory and immunosuppressive roles (Selvaraj et al., 2020; Theoharides and Conti, 2020). Steroid had been used in about in 57% of our patients.

Antibiotic is another medication that had been used in 71% of our patients based. The excess use of antibiotics in our patients is a reflection of anxiety, fear, and uncertainty surrounded the pandemic and the absence of anti-viral medication with proven efficacy. As well, AKH was the first hospital in UAE to be declared as a COVID-19 center, therefore, employee of AKH were at the frontline of fighting the first reported cases of COVID-19 in the UAE. More, fever and cough were the most common symptoms in our patients and the presence of the radiological infiltrates presented in more than 60% of the patients. Fever, cough and radiological infiltrate are hallmarks of bacterial community-acquired pneumonia which requires antibiotic treatment (Huttner et al., 2020), Hence, this contributed to excess use of antibiotics and some other medications in our COVID-19 patients.

The excess use of antibiotics is supported by the fact that literature does not indicate that antibiotics are effective in treating COVID-19 (Zhou et al., 2020), and the incidence of bacterial coinfections appears low among COVID-19 (Rawson et al., 2020). Rawson et al. reported that although among their COVID-19 patients only 8% experienced a bacterial or fungal coinfection, 72% received antibiotics. a percentage that is similar to what we found in our COVID-19 patients (Rawson et al., 2020).

Older age, hypertension and diabetes were the risk factor for ICU admission is aligned with what had published previously that showed that the same risk factors more common among ICU patients (Hachim et al., 2020) and it contribute to the development of acute cardiac injury among COVID-19 patients (Naeem et al., 2020).

### Strength and Limitations

Although the study is a single center and it lack the social determinants of the patients but it included the first cases affected by COVID-19 in the UAE and the sample size is fairly good with wide range clinical and laboratory data.

## Conclusion

Our patients are younger and mainly male, DM and hypertension were the most common comorbidities. The clinical symptoms are mainly of LRTI and there were considerable percentage of clinical complications and organ dysfunction. The treatment showed excess use of antibiotics.

## Data Availability Statement

The original contributions presented in the study are included in the article/supplementary material. Further inquiries can be directed to the corresponding author.

## Ethics Statement

The studies involving human participants were reviewed and approved by the Scientific Research Committee of MOHAP, approval letter No. MOHAP/DXB-REC/MMM/NO.44/2020. The patients/participants provided their written informed consent to participate in this study.

## Author Contributions

All authors have contributed equally to the manuscript. They all contributed in the planning, data collection, analysis, drafting and writing the article. All authors contributed to the article and approved the submitted version.

## Conflict of Interest

The authors declare that the research was conducted in the absence of any commercial or financial relationships that could be construed as a potential conflict of interest.

## Abbreviations

ARDS, Acute Respiratory Distress Syndrome; ALT, Alanine Transaminase; AST, Aspartate Aminotransferase; BMI, Body Mass Index; COVID-19, Corona Virus Disease-2019; CRP, C-Reactive Protein; CVD, Cardiovascular Disease; DM, Diabetes Mellitus; eGFRigure, Estimated Glomerular Filtration Rate; ICU, Intensive Care Unit; INR, International Normalized Ratio; IL-6, Interlukin-6; LDH, Lactate Dehydrogenase; LRTI, Lower Respiratory Tract Infection; SOB, Shortness of Breath; URTI, Upper Respiratory Tract Infection; WCC, White Cell Count.
